# WHAT KIND OF HEALTH SCREENING IS USEFUL FOR PERSONS AGING WITH HIV?

**DOI:** 10.1093/geroni/igae098.3930

**Published:** 2024-12-31

**Authors:** Manuel Ramos, Shelby Brage, Christian Nouryan, Bruce Hirsch, Joseph McGowan, Ashwin Mattam, Edith Burns

**Affiliations:** The Donald and Barbara Zucker School of Medicine at Hofstra/Northwell, Floral Park, New York, United States; The Feinstein Institutes for Medical Research, Manhasset, New York, United States; Zucker School of Medicine at Hofstra/Northwell, Manhasset, New York, United States; Zucker School of Medicine at Hofstra/Northwell, Manhasset, New York, United States; Zucker School of Medicine at Hofstra/Northwell, Manhasset, New York, United States; Zucker School of Medicine at Hofstra/Northwell, Manhasset, New York, United States; Northwell Health, Manhasset, New York, United States

## Abstract

**Background:**

In the United States, 54% of people aging with HIV (PAWH) are age >/= 50 and 89.7% are virally suppressed. HIV is linked to increased risk of developing comorbidities and may accelerate aging. This population may not receive adequate screening for changes related to advancing age. We describe initial findings of a “Maturity Screen” for PAWH.

**Methods:**

Setting and population. Convenience sample of PAWH aged 50 years or older receiving care at an HIV specialty clinic in a major metropolitan area. Patients completed a series of measures administered by trained care team members. Measures. sociodemographic (e.g., age, sex), comorbid conditions. Maturity Screen: MoCA, PHQ2, ADL, IADL, GAD2, gait speed and Edmonton Frail Scale.

**Results:**

82 participants were screened to-date; 41 males, 36 females, 4 unknown. Mean age 63 (SD: 7.6 yrs.); mean # comorbid conditions 1.8 (SD: 1.3, range 0-5); top four comorbidities were hyperlipidemia (56%), hypertension (43%), diabetes (11%), and CKD (11%). Mean MoCA 24.9 (SD: 3.7, range 15-30); mean PHQ2 0.6 (SD: 1.2); ADL 5.9 (SD: 0.5); IADL 7.8 (SD: 0.8, max score 9).

**Conclusion:**

In this sample of PAWH maturity screening suggests healthier group than anticipated and low levels of depression. Borderline mean cognitive screen suggests need to monitor for emerging issues likely to impact quality of life. Examining social support in the context of PAWH and potential cognitive decline may identify those with increased future healthcare needs and poor outcomes. Gait speed and frailty measures are currently being analyzed and may identify additional functional needs.

